# Optimal Compensation of MEMS Gyroscope Noise Kalman Filter Based on Conv-DAE and MultiTCN-Attention Model in Static Base Environment

**DOI:** 10.3390/s22197249

**Published:** 2022-09-24

**Authors:** Zimin Huo, Fuchao Wang, Honghai Shen, Xin Sun, Jingzhong Zhang, Yaobin Li, Hairong Chu

**Affiliations:** 1 Changchun Institute of Optics, Fine Mechanics and Physics, Chinese Academy of Sciences, Changchun 130033, China; 2 University of Chinese Academy of Sciences, Beijing 100049, China; 3 Key Laboratory of Airborne Optical Imaging and Measurement, Changchun Institute of Optics, Fine Mechanics and Physics, Chinese Academy of Sciences, Changchun 130033, China; 4 Forest Protection Research Institute of Heilongjiang Province, Harbin 150040, China

**Keywords:** MEMS gyroscope, convolutional denoising autoencoder, temporal convolutional network, attention mechanism, Particle Swarm Optimization algorithm, Kalman filter

## Abstract

Errors in microelectromechanical systems (MEMS) inertial measurement units (IMUs) are large, complex, nonlinear, and time varying. The traditional noise reduction and compensation methods based on traditional models are not applicable. This paper proposes a noise reduction method based on multi-layer combined deep learning for the MEMS gyroscope in the static base state. In this method, the combined model of MEMS gyroscope is constructed by Convolutional Denoising Auto-Encoder (Conv-DAE) and Multi-layer Temporal Convolutional Neural with the Attention Mechanism (MultiTCN-Attention) model. Based on the robust data processing capability of deep learning, the noise features are obtained from the past gyroscope data, and the parameter optimization of the Kalman filter (KF) by the Particle Swarm Optimization algorithm (PSO) significantly improves the filtering and noise reduction accuracy. The experimental results show that, compared with the original data, the noise standard deviation of the filtering effect of the combined model proposed in this paper decreases by 77.81% and 76.44% on the *x* and *y* axes, respectively; compared with the existing MEMS gyroscope noise compensation method based on the Autoregressive Moving Average with Kalman filter (ARMA-KF) model, the noise standard deviation of the filtering effect of the combined model proposed in this paper decreases by 44.00% and 46.66% on the *x* and *y* axes, respectively, reducing the noise impact by nearly three times.

## 1. Introduction

MEMS gyroscopes have the characteristics of small size, low power consumption, low cost, and high-cost performance [1]. It is easier to act as an actuator or a key node of inertial navigation in small institutions, such as in the drone remote sensing measurement gimbals [2], aviation pods [3,4], navigation terminals [5,6], and other institutions, and it plays an important role. High-precision MEMS gyroscopes can already meet the needs of engineers for practical projects, so reducing the noise of MEMS gyroscopes and improving measurement accuracy has become a hot issue.

Traditional gyroscope noise reduction methods include Kalman filter [7], Fast Fourier Transform [8], Empirical Mode Decomposition [9], Wavelet Transform [10], Variational Mode Decomposition [11], and Ensemble Empirical Mode Decomposition [12], etc. For example, Liu, Fuchao [13] proposed an adaptive unscented Kalman filter algorithm by analyzing the influence of the MEMS IMU noise statistical characteristics on the accuracy of the angular rate solution of a high-rotating projectile and verified that the algorithm has better performance than the unscented Kalman filter algorithm with higher estimation accuracy. Yingjie Hu [14] proposed a method combining wavelet denoising with time series analysis, using wavelet denoising to deal with high-frequency noise, followed by time series analysis combined with the Sage-Husa adaptive Kalman filter to remove low-frequency noise. Siyuan Liang [15] proposed to use the compression characteristics of multi-scale wavelet transform to compress the original signal of MEMS gyroscope, fuse the compressed data according to the support degree, and then perform threshold processing on the fused wavelet coefficients to improve the accuracy of MEMS inertial devices. The harsh environment of actual engineering often limits traditional methods, so machine learning represented by the neural network and support vector machine has also been derived to filter MEMS gyroscopes [16]. Huiliang Cao [17] utilized three methods, radial basis function neural network (RBF NN), genetic algorithm (GA)-based RBF NN, and GA-based RBF NN with Kalman filter, to effectively compensate for the temperature energy-influenced drift of MEMS vibrating gyroscopes. Rita Fontanella [18] used a back-propagation artificial neural network as an improvement of polynomial fitting in order to solve the zero bias when the polynomial was applied to temperature calibration of MEMS gyroscope, and the study applied it to the Attitude and Heading Reference System model to improve its attitude accuracy by 20%. Mitchell Webber [19] used a combination of support vector machine and Kalman filtering to fuse the data of MEMS gyroscopes and accelerometers in wearable devices to achieve noise reduction and recognition.

At this stage, in order to further increase the production cost of the equipment and improve the cost performance, designers often only use cost-effective MEMS gyroscopes instead of the optical fiber gyroscopes as essential components. With the continuous improvement of computer arithmetic power, attempts have been made to use deep learning models with more robust fitting capabilities to denoise MEMS gyroscope signals [20,21,22]. Changhui Jiang [23] proposed an artificial intelligence method for denoising the output signal of a MEMS IMU, where the signal was processed as a time series, and a long short-term memory (LSTM) was used to filter the output signal of a MEMS gyroscope. Martin Brossard [24] used convolutional neural networks to calculate gyro corrections based on past gyro measurements as a way of filtering out undesirable errors in the original gyro signal. Israr Ullah [25] used an artificial neural network-based learning module to estimate the amount of error in the sensor readings and update the measurement covariance R in the Kalman filter accordingly, resulting in a reduction in sensor noise of around 10%. It is possible to use deep learning techniques to eliminate MEMS gyroscope noise. Although the research on noise reduction of MEMS gyroscopes based on deep learning is just in its infancy, existing research shows that the noise reduction of MEMS gyroscopes based on deep learning is undoubtedly a new idea.

In order to further improve the measurement accuracy of MEMS gyroscopes, this paper proposes an error compensation method based on the combination of Conv-DAE and MultiTCN-Attention model, then using the Kalman filter, which has the particle swarm optimization algorithm to dynamically adjust the predicted value of the combined network to improve the performance of error compensation. The main contributions of this paper are as follows:

(1) In the presence of corrupted sensor data, the feasibility of the convolutional denoising autoencoder to recover and reconstruct the signal is verified.
(2) Explore pertinent input data step sizes and network topologies to compare the error compensation performance of multilayer temporal convolutional neural (TCN) networks, their variants, and other recurrent neural network variants.
(3) The particle swarm optimization algorithm is used for parameter estimation when designing the Kalman filter. This is compared with the ARMA-KF model to further improve the filtering effect.

The rest of the paper is organized as follows: Section 2 introduces the convolutional denoising autoencoder, the temporal convolutional network, the attention mechanism, the Kalman filter, the ARMA-KF model, and the PSO-KF model, and explains the methods proposed in this paper. Section 3 presents the experiments, results, and comparisons. The rest of the paper contains conclusions and references.

## 2. Methods

This section clarifies the methods and principles proposed in the article and provides corresponding theoretical support for the subsequent experimental verification.

### 2.1. Data Reconstruction Based on Convolutional Denoising Auto-Encoder

**Figure 1:**
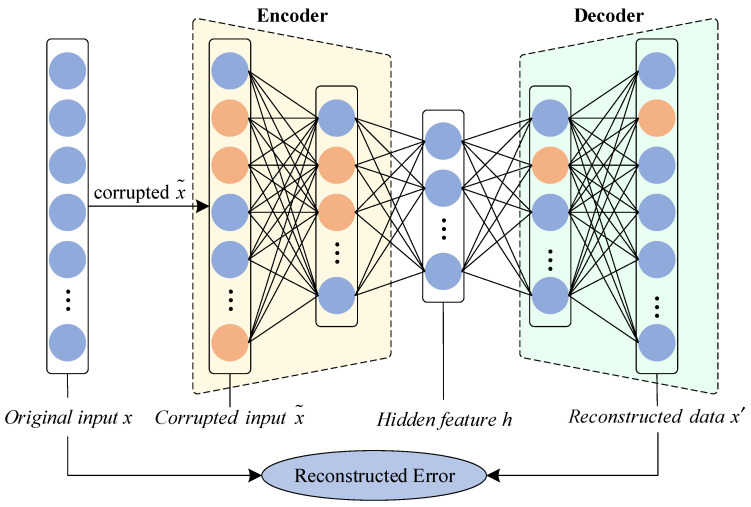
The overall structure of a denoising autoencoder.

The convolutional denoising autoencoder (Conv-DAE) model consists of an encoder and a decoder; the encoder is responsible for quickly compressing the original signal dimension and mapping it to a feature representation in low-dimensional feature space; the decoder is responsible for reconstructing this feature representation and reducing it to the original signal, the basic structure of which is shown in Figure 1 [26].

The Conv-DAE model enables efficient and accurate feature extraction of the original signal in feature space by minimizing the error between the noisy or corrupted original signal and the reconstructed original signal [27]. Compared to conventional DAEs, Conv-DAE has the same basic structure of an encoder and decoder but replaces the fully connected layers with convolutional layers. As deep-structured convolutional neural networks (CNNs) are easy to train, Conv-DAE, as a particular type of CNN, can improve the reconstruction capability by using deep structure [28,29].

**Figure 2:**
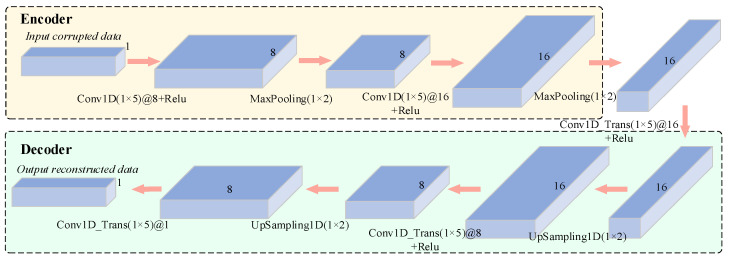
The detailed structure of the denoising autoencoder used in the experiment.

The Conv-DAE model structure proposed in this paper is shown in Figure 2 below. The model has a symmetric structure of encoder and decoder, where the encoder consists of two convolutional layers and two max-pooling layers, and the decoder consists of three transposed convolutional layers and two upsampling layers. Each convolutional layer in the encoder uses a 1 × 5 filter to extract the various feature vectors, and each transposed convolutional layer in the decoder also uses a 1 × 5 filter to reduce and aggregate the feature vectors. Details of the structure are shown in Table 1.

The convolutional layer, the max-pooling layer, the transposed convolutional layer, and the upsampling layer are the main structures for feature extraction in the Conv-DAE model proposed in this paper, with the following operational equations:

(1) $$x_{j}^{l}=f\left(\sum_{i\in M_{j}}\omega_{ij}^{l}*x_{i}^{l-1}+b_{j}^{l}\right)$$

(2) $$x_{j}^{k}\left(n\right)=\max\left[x_{j}^{k}(2n-1),x_{j}^{k}(2n)\right]$$

In Equation (1), $x_{j}^{l}$ is the current convolutional layer output features, $x_{i}^{l-1}$ is the previous layer output features, f(⋅) function is the activation function, $\omega_{ij}^{l}$ is the current convolutional layer convolutional kernel, ∗ denotes convolution, $M_{j}$ is the connection between $x_{j}^{l}$ and output features of previous layer, and $b_{j}^{l}$ is the current convolutional layer corresponding bias. In Equation (2), $x_{j}^{k}\left(n\right)$ is the *j*th convolutional kernel of the *k*th layer, *n* is the edge length of the convolutional kernel size, and max is the maximum function. In addition, the transposed convolutional layer in the decoder can be regarded as the inverse process of the convolutional layer in the encoder [30].

### 2.2. Model Prediction Based on Temporal Convolutional Networks and Attention Mechanisms

#### 2.2.1. Deep Neural Networks with Temporal Convolutional Neural Layers

**Figure 3:**
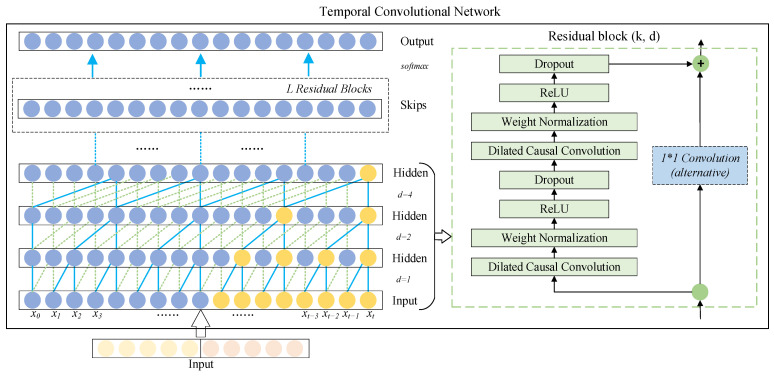
Basic structure of temporal convolutional networks.

The temporal convolutional network (TCN) [31] is primarily a temporal model based on convolutional neural networks. Unlike standard convolutional neural networks, TCN employs causal convolution for processing time series data and uses dilated convolution to cope with the long-distance dependency problem common in time series models. The basic structure of a temporal convolutional network consists of causal convolution, dilated convolution, and residual connections, as shown in Figure 3.

(a) Causal Convolution

Causal convolution is a fundamental architecture of temporal convolutional networks, and Figure 3 shows the structure of a causal convolution stack. For a one-dimensional time series input $X=(x_{0},x_{1},\ldots ,x_{t},\ldots ,x_{T})$, the output $y_{t}$ of time *t* depends only on the current time $x_{t}$ and partial past time input (i.e., $x_{t-1},x_{t-2},x_{t-3},\ldots ,x_{t}$), not any future input (i.e., $x_{t+1},x_{t+2},x_{t+3},\ldots ,x_{T}$). Therefore, the output information of the temporal convolutional network is only affected by the past input information, avoiding the “leakage” that never came in the past. In addition, causal convolution is susceptible to the limitations of the receptive field, i.e., the output can only be predicted by receiving information from a shorter history size [32].

(b) Dilated Convolution

The traditional convolution operation process involves convolving the sequence once and then pooling it to reduce the sequence’s size and expand the receptive field’s size. One of its main disadvantages is that some sequence information will be lost during the pooling process. In contrast, dilated convolutions feature no pooling process but gradually increase the perceptual length through a series of dilated convolutions, so that the output of each convolution contains rich information for long-term tracking [33]. Therefore, dilated convolutions can be well applied to long information-dependent problems of sequences, such as speech and signal processing, weather forecasting, etc. For a one-dimensional time series input $X=(x_{0},x_{1},\ldots ,x_{t},\ldots ,x_{T})$ and a filter ℱ:{0,1,2,…,m−1}, the H(⋅) of the sequence element *T* of the dilated convolution operation is defined as follows:

(3) $$H\left(T\right)=\left(X{*}_{d}ℱ\right)\left(T\right)=\sum_{i=0}^{m-1}ℱ\left(i\right)\cdot x_{T-d\cdot i}$$

where *m* denotes the filter size, *d* denotes the dilation factor, ∗ denotes convolution, and T−d⋅i denotes the past direction.

The dilation operation can be thought of as introducing a fixed step between every two adjacent filters. Each layer consists of a set of dilated convolutions with rate parameters $d_{l}$, a non-linear activation $f_{nl}\left(\cdot \right)$, and a residual connection combining the input and convolution signals of the layer. $d_{l}$ represents increasing the number of consecutive layers within the block, calculated by $d_{l}=2^{l}$. The convolution operation only works between two timestamps *t* and $t-d_{l}$. Specifically, the filters can be parameterized by a weight matrix $W=\left[W_{0},W_{1}\right]$, and a bias vector *b*, where $W_{i}\in {\mathbb{R}}^{F_{w}\times F_{w}}$, $b\in {\mathbb{R}}^{F_{w}}$, and $F_{w}$ represent the number of filters.${\tilde{Z}}_{t}^{\left(j,l\right)}$ and $Z_{t}^{\left(j,l\right)}$ are the results of the null convolution and the addition of the residual join at time series *t*, respectively, denoted as

(4) $${\tilde{Z}}_{t}^{\left(j,l\right)}=f_{nl}\left(W_{0}{\tilde{Z}}_{t-d_{l}}^{\left(j,l-1\right)}+W_{1}{\tilde{Z}}_{t}^{\left(j,l-1\right)}\right)$$

(5) $$Z_{t}^{\left(j,l\right)}=Z_{t}^{\left(j,l-1\right)}+V{\tilde{Z}}_{t}^{\left(j,l\right)}+e$$

where $V\in {\mathbb{R}}^{F_{w}\times F_{w}}$ denotes the weight matrix, and $e\in {\mathbb{R}}^{F_{w}}$ denotes the bias vector of residual connections [34].

(c) Residual Connections

Residual connections have proven to be an effective method to train deep networks, which allow the network to pass information across layers [31,33]. In addition, the receptive field size of TCN can be enlarged by changing the number of hidden layers in residual connections, and the problem of vanishing gradients in the process of training neural networks can be avoided.

One branch of the residual block performs the transformation operation G(⋅) on the input $X^{\left(h-1\right)}$, and a branch is added to perform a straightforward transform to keep the number of feature maps in parallel with the existing branches. The output $X^{\left(h\right)}$ of the *h*th residual block can be expressed as:

(6) $$X^{\left(h\right)}=\delta \left(G\left(X^{\left(h-1\right)}\right)+X^{\left(h-1\right)}\right)$$

where δ(⋅) indicates the activation operation. G(⋅) is a series of transformation operations. As shown in the right half of Figure 3, the residual connection structure includes dilated causal convolutional layers, weightnorm layers, activation layers, and dropout layers. Among them, the dilated causal convolution layer is composed of the aforementioned causal convolution and dilated convolution, which is used to extract hidden features from the input; the weightnorm layer is used to improve the training speed by limiting the weight range; the activation layer adopts a good convergence Rectified Linear Unit (ReLU); and the dropout layer is used for regularization to solve the overfitting problem of deep networks.

Therefore, in contrast to long short-term memory and the gated recurrent neural network, (1) TCN can perform convolution in parallel due to its parallelism; (2) TCN can adjust the receptive field size by the number of layers, dilation factor, and filter size, which allows us to control the memory size of the model for different domain requirements; (3) in the depth direction of the network, since TCN uses residual connections when the input length is very long, the gradient in TCN will have more robust stability. Based on the above characteristics, the temporal convolutional network can effectively avoid the gradient disappearance or gradient explosion problem of the recurrent neural networks.

#### 2.2.2. Attention Mechanism

**Figure 4:**
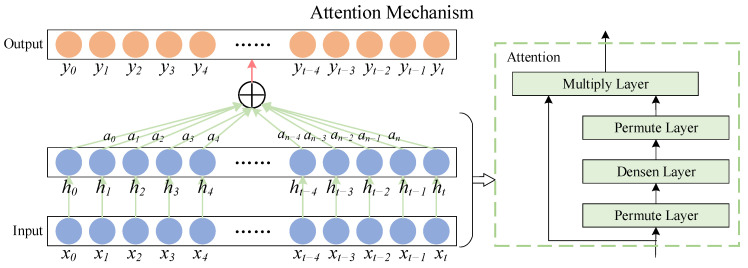
Attention mechanism structure diagram.

The attention mechanism is a simulation of the human brain’s form of assigning attention, and its essence is to change the weight of features in the hidden layer [35]. The attention mechanism can reasonably filter out a small number of critical features from a large number of features and assign more weight to them, reducing the weight of non-key features to highlight the impact of critical features. Fusing attentional mechanisms with temporal convolutional networks can highlight key features and improve prediction accuracy. The structural principle of the attentional mechanism is shown in Figure 4.

Where $x_{t}\left(t\in \left[0,n\right]\right)$ is the input to the deep neural network, $h_{t}\left(t\in \left[0,n\right]\right)$ corresponds to the hidden layer output obtained by passing each input through the deep neural network, $a_{t}\left(t\in \left[0,n\right]\right)$ is the attention weight of the attention mechanism on the hidden layer output of the deep neural network, and $y_{t}\left(t\in \left[0,n\right]\right)$ is the output value of the attention mechanism introduced. The calculation formula of the weight coefficient of the attention mechanism can be expressed as:

(7) $$e_{t}=u\tanh\left(wh_{t}+b\right)$$

(8) $$a_{t}=\frac{\exp\left(e_{t}\right)}{\sum_{j=0}^{t}e_{j}}$$

(9) $$y_{t}=\sum_{t=0}^{n}a_{t}h_{t}$$

where $e_{t}$ represents the attention weight determined by the output layer vector $h_{t}$ of the deep neural network at time *t*, *u* and *w* are the weight coefficients; *b* is the bias coefficient, and $y_{t}$ is the output of the attention layer at time *t*. The attention mechanism automatically calculates the corresponding weight assignments for the in-depth features and merges them into a new vector. The input to this layer is the output vector of the deep neural network, the Permute layer rearranges the dimensions of the input according to a given pattern, and the Multiply layer completes the output of the attention with the output of the deep neural network by multiplying the output bit by bit, achieving a dynamic weighting process for the hidden layer units, and thus highlighting the impact of critical features on the final result [36].

### 2.3. Multi-Layer Deep Learning Network Combination Model

**Figure 5:**
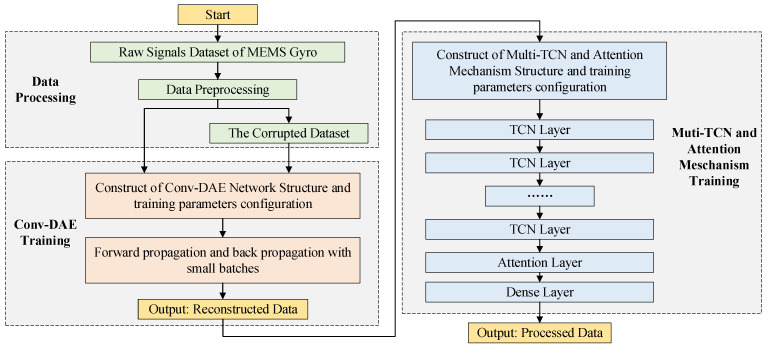
Multi-layer deep learning network combination model structure diagram.

In order to further improve the prediction performance of the MultiTCN-Attention model, this article proposes a method based on the combination of convolutional denoising autoencoder and MultiTCN-Attention model. After data reconstruction is carried out through the convolutional denoising autoencoder model, the output result is used as the input of the MultiTCN-Attention model for prediction processing. The specific structure and parameter configuration of the convolutional noise reduction autoencoder model are described in Section 2.1 of this paper. When the MEMS gyroscope is sampled for a long time, due to the limitation of the communication between the MEMS gyroscope and the host computer equipment, packet loss will occur. Therefore, in order to imitate the appearance of this phenomenon, 5% of the original MEMS gyroscope data are randomly damaged and reset, as the input data of the convolutional denoising autoencoder and the original data are compared. The data reconstruction operation is performed, as shown in Figure 5. The reconstructed data output by the convolutional denoising autoencoder is used as the input to the next model.

The MultiTCN-Attention model was chosen to build multi-layer TCN, and the addition of an attention mechanism layer made the multi-layer TCN more focused on what was beneficial to the outcome. The output layer was a fully connected layer that accepted the output vector from the attention mechanism weighted processing and processed it into the predicted value of the MEMS gyroscope. The detailed parameter configuration of the MultiTCN-Attention model is described in a later section. As can be seen, the input vector starts at the input layer and it is processed by several TCN layers before entering the attention mechanism, which calculates the attention weight vector based on the current input vector and merges the two to obtain a new vector, which is fed into the fully connected layer to output the predicted value.

### 2.4. Particle Swarm Optimization Algorithm for Optimal Kalman Filter and Others

The Kalman filter is a recursive filter (autoregressive filter) capable of estimating the state of a dynamic system from a series of incomplete and noise-containing measurements by considering the joint distribution at each time based on the values of each measurement at different times, thus producing an estimate of the unknown variables [37]. Kalman filtering mainly includes two parts: the prediction process and the update process. It is assumed that the state-space model of the system (state equation and measurement equation) is as follows:

(10) $$x_{k}=\Phi_{k/k-1}x_{k-1}+B_{k-1}w_{k-1}$$

(11) $$y_{k}=H_{k}x_{k}+v_{k}$$

where $x_{k}$ is the system state vector, $\Phi_{k/k-1}$ is the system state transition matrix, $B_{k-1}$ is the system noise driving matrix, and $w_{k-1}$ is the state excitation noise or system noise; $y_{k}$ is the measurement vector, $H_{k}$ is the measurement matrix, and $v_{k}$ is the measurement noise. Moreover, assume that $w_{k},v_{k}$ are Gaussian white noise sequences with zero mean, and the two white noises are uncorrelated with each other, satisfying:

(12) $$\{\begin{array}{c} E[w_{k}]=0,\\ E[v_{k}]=0,\\ E[w_{j}v_{k}^{T}]=0, \end{array}\begin{array}{c} E[w_{j}w_{k}^{T}]=Q_{k}\delta_{jk}\\ E[v_{j}v_{k}^{T}]=R_{k}\delta_{jk} \end{array}\left(Q_{k}>0,R_{k}>0\right)$$

In the prediction process, the current system state vector is predicted from the previous moment’s system state vector such that:

(13) $${\hat{x}}_{k/k-1}=\Phi_{k/k-1}{\hat{x}}_{k-1}$$

(14) $$P_{k/k-1}=\Phi_{k/k-1}P_{k-1}\Phi_{k/k-1}^{T}+B_{k-1}Q_{k-1}B_{k-1}^{T}$$

where ${\hat{x}}_{k/k-1}$ is the predicted value of the system state vector, and $P_{k/k-1}$ is the predicted covariance matrix of the system state vector.

In the update process of the Kalman filter, the current system state vector is updated with the measurement vector such that:

(15) $$K_{k}=P_{k/k-1}H_{k}^{T}{\left(H_{k}P_{k/k-1}H_{k}^{T}+R_{k}\right)}^{-1}$$

(16) $${\hat{x}}_{k}={\hat{x}}_{k/k-1}+K_{k}\left(y_{k}-H_{k}{\hat{x}}_{k/k-1}\right)$$

(17) $$P_{k}=P_{k/k-1}-K_{k}H_{k}P_{k/k-1}$$

where $K_{k}$ is the Kalman filter gain matrix, and $P_{k}$ is the updated covariance matrix of the system state vector.

#### 2.4.1. Kalman Filter Based on ARMA Model

The Autoregressive Moving Average (ARMA) model is obtained by regressing the dependent variable on its lagged values as well as the present and lagged values of the random error term [38]. Moreover, it is one of the standard methods used in time series analysis. The ARMA model can be expressed as follows:

(18) $$x_{k}=\sum_{i=1}^{p}\varphi_{i}x_{k-i}+\sum_{j=1}^{q}\theta_{j}\varepsilon_{k-j}+\varepsilon_{k}$$

(19) $$\varepsilon_{k}∼W\left(0,\sigma^{2}\right)$$

That is, the autoregressive moving average model ARMA (*p*, *q*). *p* and *q* are the acceptance orders of the autoregressive (AR) and moving average (MA) models, respectively. In addition, *p* is also expressed as the number of lags in the time series data itself used, and *q* represents the number of forecast error lags used in the forecast model. They are determined by the nature of the time series data itself. $x_{k}$ is the observed time series data; $\varepsilon_{k}$ represents a discrete white noise sequence with mean 0 and variance $\sigma^{2}$. $\varphi_{i}<1\left(i=1,2,\ldots ,p\right)$ is the autoregressive coefficient, and $\theta_{j}<1\left(j=1,2,\ldots ,q\right)$ is the moving average coefficient.

The steps for designing the Kalman filter using the ARMA model are as follows [39,40,41]: (1) data pre-processing, including the removal of wild values, removal of constant components and extraction of trend terms, and data testing; (2) determination of the model type based on the autocorrelation function and partial autocorrelation function; (3) determination of the order based on the Akaike Information Criterion; and (4) adaptive testing of the designed model.

#### 2.4.2. Particle Swarm Optimization Algorithm for Optimal Kalman Filter

In order to further improve the accuracy of the Kalman filter, in addition to using the traditional ARMA time series modeling, this paper chooses to optimize the parameters of the Kalman filter using the particle swarm optimization algorithm. Particle swarm optimization has attracted more researchers because of its flexibility and robustness, especially for problems in dynamic environments. PSO is a swarm-based stochastic optimization technique inspired by social behaviors such as bird flocking or fish flocking [42].

**Figure 6:**
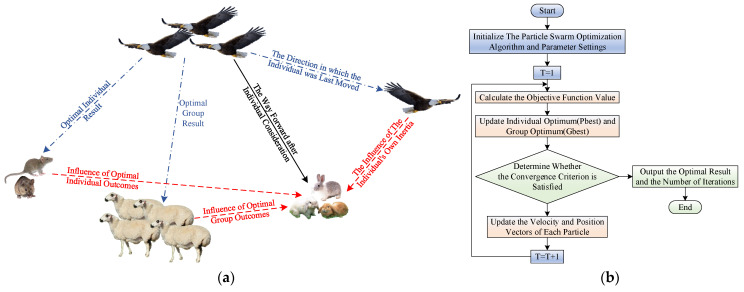
Particle swarm optimization algorithm: (**a**) the basic principle diagram of PSO; (**b**) basic flow chart of PSO.

As shown in Figure 6a, suppose a flock of birds is randomly searching for food. Additionally, suppose a piece of food that is known to be in a particular area, but none of the birds know exactly where it is. However, they can use their own experience (optimal individual choice) and group experience (optimal global choice) to predict how far away the current location is from the food to find the location of the food quickly [43]. This bird predation mechanism inspires the particle swarm optimization algorithm, so the basis of PSO is the group sharing of information.

The particle swarm optimization algorithm consists of a large swarm of particles in which *n* particles fly in the *D*-dimensional space. Each particle maintains the particle position $x_{i}$, the direction and speed of particle movement $v_{i}$, and the searched optimal position fitness value $p_{i}$ in the *D*-dimensional space, which can be expressed as:

(20) $$\{\begin{array}{c} x_{i}=\left(x_{i1},x_{i2},x_{i3},\ldots ,x_{iD}\right)\\ v_{i}=\left(v_{i1},v_{i2},v_{i3},\ldots ,v_{iD}\right)\\ p_{i}=\left(p_{i1},p_{i2},p_{i3},\ldots ,p_{iD}\right) \end{array}$$

The improvement of the flying speed, position, and weight of particle *i* can be adjusted according to Equations (21)–(23).

(21) $$v_{id}^{k+1}=\omega v_{id}^{k}+c_{1}rand\left(p_{ib}-x_{ib}^{k}\right)+c_{2}rand\left(p_{gb}-x_{ib}^{k}\right)$$

(22) $$x_{ib}^{k+1}=x_{ib}^{k}+v_{ib}^{k}$$

(23) $$\omega =\omega_{\max}-\left(\omega_{\max}-\omega_{\min}\right)\frac{iter}{iter_{\max}}$$

In the formula, *d* and *k* represent the dimension and the number of iterations, respectively; *b* represents the *b*th generation. $p_{ib}$ represents the best position of particle *i*, $p_{gb}$ represents the current best position; $c_{1}$ and $c_{2}$ represent the individual learning factor and group learning factor, respectively; rand(⋅) is used to obtain random values in the range of [0,1]. ω is the inertia weight used to balance the global search ability and local search ability, which can be updated iteratively by using Equation (23); $\omega_{\max},\omega_{\min}$ are the maximum and minimum inertia weights, respectively; $iter,iter_{\max}$ are the current and maximum number of iterations, respectively.

**Figure 7:**
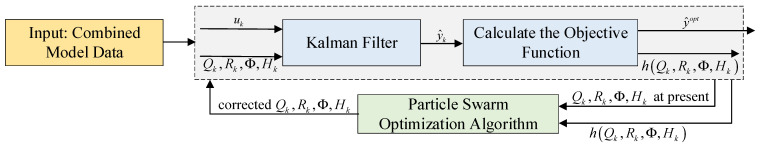
Structure of Kalman filter based on particle swarm optimization.

As shown in Figure 7, when optimizing the four parameters $Q_{k},R_{k},\Phi ,H_{k}$ of the Kalman filter using the particle swarm optimization algorithm, according to the Formula (16) in the update process of the Kalman filter, avoiding premature convergence of the optimization seeking process to be able to obtain the optimal global solution, the actual variance of the innovation is selected here as the objective function, with its value minimized as the objective for optimization. The specific PSO process is shown in Figure 6b. Define the objective function as shown in Equations (24) and (25):

(24) $$h={\hat{y}}_{k/k-1}{\left({\hat{y}}_{k/k-1}\right)}^{T}$$

(25) $${\hat{y}}_{k/k-1}=y_{k}-H_{k}{\hat{x}}_{k/k-1}$$

Among them, *h* is the actual variance of the state information, and ${\hat{y}}_{k/k-1}$ is the innovation sequence generated by the Kalman filter [44].

## 3. Validation of the Proposed Method

In this section, the method proposed in the article was tested, the corresponding experimental design and result analysis were given, and the method’s validity was verified.

### 3.1. Acquisition of Test Data

**Figure 8:**
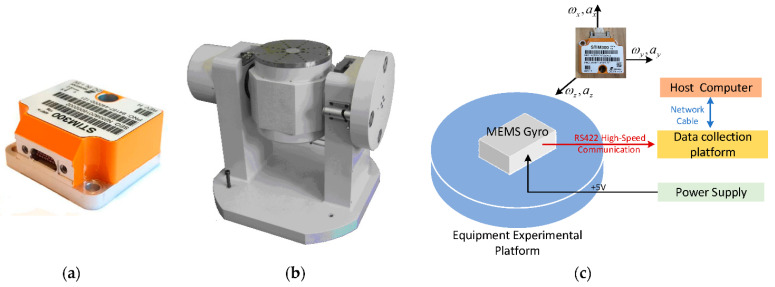
Equipment testing device. (**a**) STIM300 IMU; (**b**) static IMU data collection system; (**c**) data acquisition procedure.

This article used the STIM300 IMU (Safran Sensing Technologies, Horten, Norway) as the measured object, composed of a three-axis MEMS gyroscope, a three-axis MEMS accelerometer, and a three-axis MEMS inclinometer. The physical drawing and gyroscope specifications of the STIM300 are shown in Figure 8a and Table 2, respectively. The STIM300 was fixed to a high-precision static base stage, as shown in Figure 8b. The data acquisition flow of the STIM300 is shown in Figure 8c. The data from the STIM300 were sent to the xPC via the RS422 communication interface at a baud rate of 921,600 bps. xPC decoded the gyroscope data and sent them to the host computer via the network cable. The STIM300 gyroscope was powered up firstly and then preheated for 20 min at room temperature. Lastly, static test experiments were performed.

**Figure 9:**
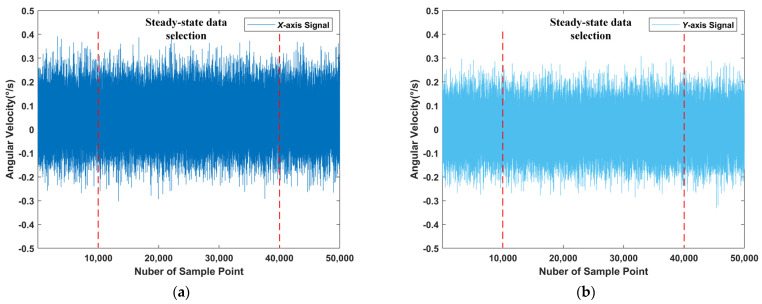
Raw gyroscope data under static conditions. (**a**) *X*-axis raw signal; (**b**) *Y*-axis raw signal.

In order to adapt to the application scenario of the STIM300 gyroscope, the platform to which the gyroscope equipment was adapted was mainly used to measure the pitch angular velocity and yaw angular velocity of the photoelectric stabilization platform. As shown in Figure 8c, the pitch angle was obtained by rotating the plane YOZ with the *y*-axis, and the yaw angle was obtained by rotating the plane XOZ with the *x*-axis. Therefore, we mainly studied the *x*-axis and *y*-axis angular velocity. The static raw data obtained from the measurement are shown in Figure 9.

### 3.2. Comparison of Training Based on Convolutional Denoising Auto-Encoders

In order to further apply the deep learning model and the construction of the ARMA model, this paper chose to use the pre-data processing method of the ARMA model to process the raw data, mainly including the elimination of wild values, the removal of constant components, and the extraction of trend terms [39,40,41]. To consider model generality and accuracy, we took the first 80% of the processed *x*-axis and *y*-axis data as the training set and the last 20% of the *x*-axis and *y*-axis data as the test set.

The deep learning algorithms proposed in this paper were performed on Tensorflow 2.3.0 (Google, Mountain View, CA, USA) and Keras 2.4.3 (Google, USA) running on Ubuntu 16.04-LTS-x86 64-bit operating system (Canonical Ltd., London, UK). The computer platform was equipped with Intel i7-4770 CPU (Intel, Santa Clara, CA, USA), 16G memory (SKhynix, Icheon-si, Korea), 2T SSD (Samsung, Seoul, Korea), and GeForce RTX-2080Ti GPU (NVIDIA, Santa Clara, CA, USA). In order to compare the superiority of convolutional denoising autoencoders, this paper used the normal denoising autoencoder (Normal-DAE) listed in Table 3 to compare with the convolutional denoising autoencoders listed in Table 1 above. They adopted the Adam optimization algorithm for updating network parameters, using mean squared error (MSE) as the loss function.

The preprocessed *x*-axis and *y*-axis data volume of 30,000 were used as the input number of the denoising autoencoder, and the randomly damaged data were set to account for 5% of the total data volume. The batch_size was set to 200, the number of epoch was set to 100, and input_size was set to (20,1) for deep learning training.

**Figure 10:**
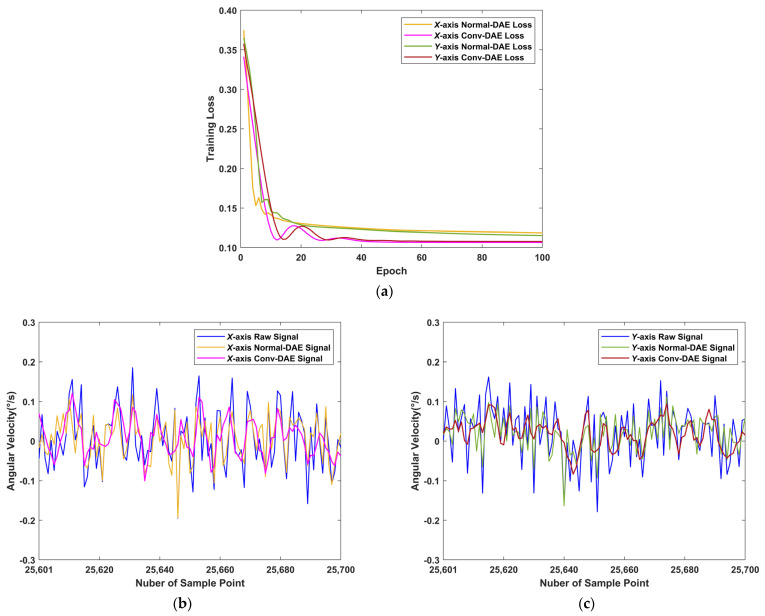
Denoising autoencoder training results: (**a**) DAE training loss; (**b**) *X*-axis raw DAE results; (**c**) *Y*-axis raw DAE results.

The results of the convolutional denoising autoencoder are shown in Table 4 and Figure 10. The noise standard deviation of the MEMS gyroscope signals from the *x*-axis and *y*-axis decreased by approximately 23.41% and 28.72%, respectively, after processing by the normal denoising autoencoder, while the noise standard deviation of the signals decreased by approximately 44.63% and 38.44%, respectively, after processing by the convolutional denoising autoencoder proposed in this paper. It can be shown that the proposed convolutional denoising autoencoder outperformed normal denoising autoencoder in terms of noise reduction and signal reconstruction of MEMS gyroscope signals. It prepared the signals processed by the convolutional denoising autoencoder for further processing in the later paper.

### 3.3. The Training Based on Combinatorial Model Compared with Other Neural Networks

To validate the performance of the MultiTCN-Attention model for gyroscope error compensation in the static base environment, this paper used data reconstructed by the convolutional denoising autoencoder as the input values for deep learning. The MultiTCN model was first explored using an *x*-axis test set with appropriate values for the input data step size, number of hidden cells, number of hidden layers, and dilation list, with the base settings shown in Table 5, and it took the Adam optimization algorithm and mean squared error (MSE) loss function. Subsequently, the training was carried out using the determined values. The MultiTCN-Attention network results were compared with MultiTCN networks and LSTM networks using the *x*-axis and *y*-axis test sets, respectively. As shown in Table 6, Table 7, Table 8 and Table 9, when the input data stride and the number of hidden layers were wider, the training time per epoch was longer. We need to make a trade-off between results and computational performance. According to the comparisons, the best results were obtained when the input data stride was 20, the number of hidden units was 128, and the number of hidden layers was 4. While this did not indicate that this was an optimal parameter for the network, it would be an appropriate value given the computational resources.

**Figure 11:**
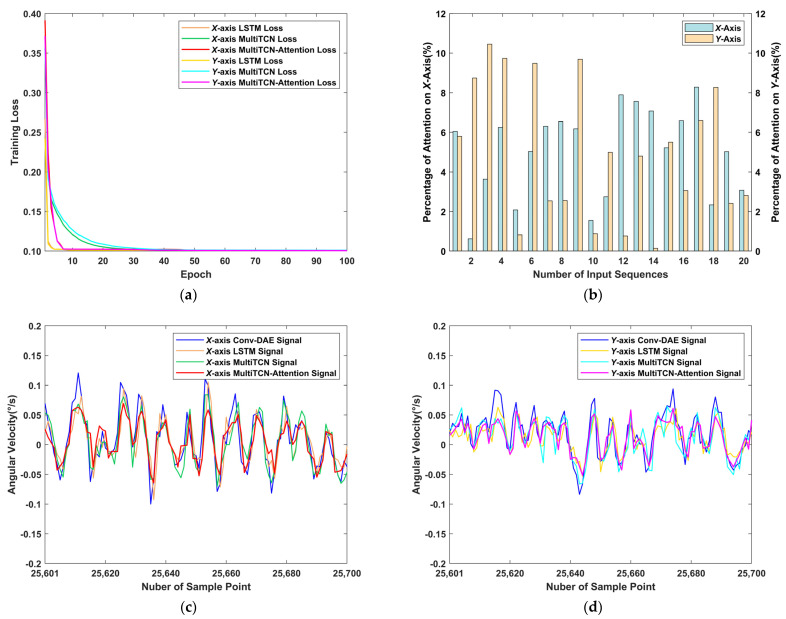
MulitTCN-Attention and other neural network training results: (**a**) training loss; (**b**) the percentage of attention mechanism; (**c**) *X*-axis training results; (**d**) *Y*-axis training results.

For the MultiTCN-Attention model, we set the following parameters according to the above conclusions, as shown in Table 10. The attention layer was set to the same length as the input length, and the results are shown in Figure 11 and Table 11 and Table 12. Figure 11a shows the training losses within 100 epochs and convergence is achieved for all networks; Figure 11b shows the weights of the sequence output values in the total sequence as calculated by the attention mechanism; as shown in the figure for the *x* and *y* axes, the distribution of attention is different, with more even attention on the *x*-axis and more focused attention on the front of the sequence input for the *y*-axis. Table 11 and Table 12 show that the MultiTCN-Attention model resulted in a 58.15% and 57.89% reduction in the standard deviation of noise in the *x* and *y* axes, respectively, compared to the raw data, proving that the application of the MultiTCN-Attention model in MEMS gyroscope error compensation studies was feasible. In addition, compared with the results of the LSTM and the MultiTCN, the noise standard deviation values of the MultiTCN-Attention model results on the *x*-axis were reduced by 11.68% and 9.46%, respectively, and the deviation values on the *y*-axis were reduced by 17.05% and 9.52%, respectively. This indicated that the MultiTCN-Attention model outperformed both networks regarding error compensation.

### 3.4. Optimization of Kalman Filter Parameters Based on Particle Swarm Optimization Algorithm and Others

In this section, the raw data and MultiTCN-Attention combined model results on the *x*-axis and *y*-axis were used as measurements, respectively. The parameters of the Kalman filter were estimated by the ARMA model and particle swarm optimization algorithm, and the filtering results were compared.

In order to make the experimental data more extensive and adaptable, the data of the MultiTCN-Attention combined model were no longer analyzed using the ARMA model method in this paper, only the particle swarm optimization algorithm was used to optimize the parameters of the Kalman filter, and the raw data were analyzed using the PSO-KF method and the ARMA-KF method.

#### 3.4.1. Determination of Kalman Filter Parameters Based on ARMA Model

In this paper, the Akaike Information Criterion was used to determine the order of the ARMA (*p*, *q*) model. If the order increases, the identified model will be more realistic, but the computational difficulty will also increase with the order increase [45]. Therefore, the maximum order was set to 3, i.e., the maximum value of *p* and *q* was set to 3. The results were as follows:

For the raw *x*-axis data, the identified model was identified as ARMA (3,2):

(26) $$x_{k}=0.0497x_{k-1}+0.7058x_{k-2}-0.0131x_{k-3}+\varepsilon_{k}-0.0534\varepsilon_{k-1}-0.6982\varepsilon_{k-2}$$

For the raw *y*-axis data, the identified model was identified as ARMA (2,2):

(27) $$x_{k}=-0.4143x_{k-1}-0.7462x_{k-2}+\varepsilon_{k}+0.4105\varepsilon_{k-1}+0.7526\varepsilon_{k-2}$$

where $x_{k}$ was the output of the ARMA model and $\varepsilon_{k}$ was the driving white noise (mean 0, variance ${\hat{\delta }}_{\varepsilon }^{2}$). The Kalman filter parameters are shown in Table 13. R is the covariance of the measurement. The initial values of the Kalman filter were set as follows: $x_{1}=\left[0;0;0;0\right]$, $P_{1}$ was the fourth-order identity matrix.

#### 3.4.2. Optimization of Kalman Filter Parameters Based on Particle Swarm Optimization Algorithm

In this paper, the particle swarm optimization algorithm was used to optimize the Kalman filter parameters, using the data and original data of the MultiTCN-Attention combined model. The optimization process was as follows (see Algorithm 1):
**Algorithm 1:** Kalman Filtering optimal solution**Input:** A numeric sequence of sensor data;**Begin:**(1)Initialize a population of particles (population size N), including random positions, weights, and velocities.(2)Evaluate the fitness of each particle according to Equations (24) and (25).(3)For each particle, compare its fitness value with the best position $p_{ib}$ it passed through; if better, use it as the current best position $p_{ib}$.(4)For each particle, compare its fitness value with the global best position $p_{gb}$ it passed through; and if better, take it as the global best position $p_{gb}$.(5)Adjust the particle velocity and position according to Equations (21)–(23).(6)Turn to step (2) if the end condition is not reached.**Output:** The Optimized parameter $\Phi_{opt},H_{opt},Q_{opt},R_{opt}$ and the Filtered Sequence.

**Figure 12:**
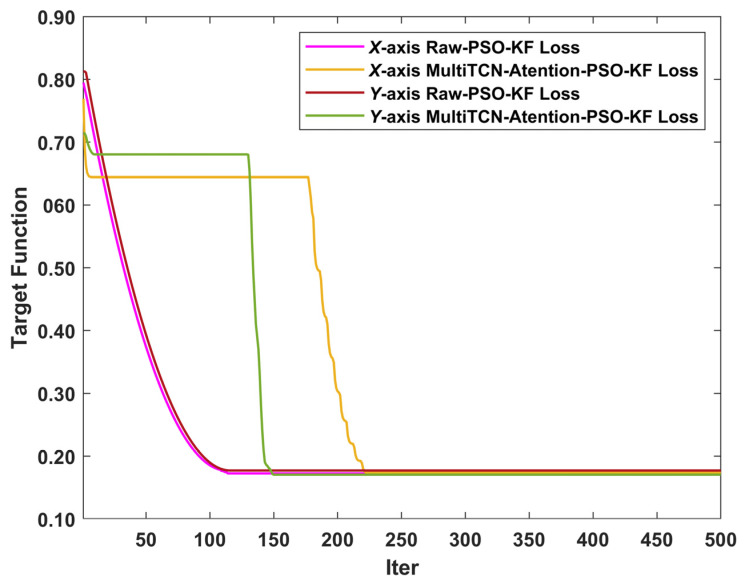
Iterative process of particle swarm optimization algorithm.

The initial values of the Kalman filter were set to $x_{1}=0$ and $P_{1}=1$, and the initial parameters were set to $\Phi_{1}=1$, $H_{1}=1$, $Q_{1}=1$, and $R_{1}=1$. The initial parameters of the particle swarm optimization algorithm were set to N=50, $iter_{\max}=500$, $\omega_{\min}=0.3$, $\omega_{\max}=0.4$, $c_{1}=0.5$, and $c_{2}=0.6$. The iterative process of the particle swarm optimization algorithm is shown in Figure 12. The parameter estimation results are shown in Table 14.

#### 3.4.3. Comparison of Kalman Filter Results

**Figure 13:**
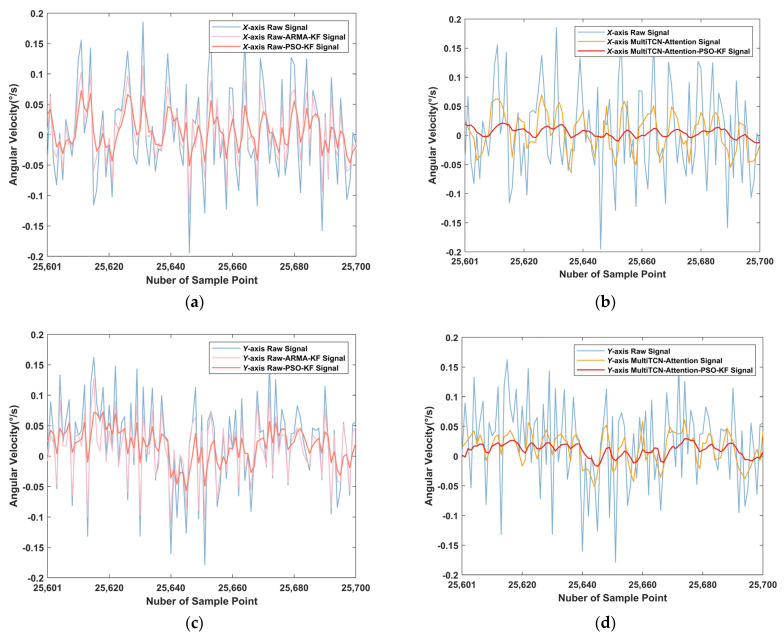
Kalman filtered effect: (**a**) *X*-axis raw data filtering results; (**b**) *X*-axis combined model filtering results; (**c**) *Y*-axis raw data filtering results; (**d**) *Y*-axis combined model filtering results.

The Kalman filtering results in this paper were shown in Table 15 and Table 16. On the *x*-axis, compared with the original data, the Kalman filtering noise standard deviation based on the particle swarm optimization algorithm was reduced by 59.65%, and the data using the MultiTCN-Attention-PSO-KF model were reduced by 77.81%, which was 25.84% and 44.71%, respectively, compared with the traditional ARMA-KF noise reduction process. On the *y*-axis, the Kalman filter noise standard deviation based on the particle swarm optimization algorithm was reduced by 59.66%, and the data using the MultiTCN-Attention-PSO-KF model were reduced by 76.44%, which was 29.88% and 46.66%, respectively, compared with the traditional ARMA-KF noise reduction process. It can be seen that the combined algorithm proposed in this paper can effectively compensate for MEMS gyroscope noise. At the same time, it can be seen from Figure 13 that the filtering effect of the combined algorithm proposed in this paper was smoother, and the signal fluctuation of the MEMS gyroscope was slight, which was closer to the actual value tested in the static base.

## 4. Conclusions

This paper proposed a combined method combining multiple neural networks and Kalman filters for MEMS gyroscope error compensation in the static base environment. By comparing the results, the following conclusions were drawn:

(1) This paper verified the feasibility of the convolutional denoising autoencoder to recover and reconstruct the signal when the sensor data were damaged and provided a new idea for signal repair.
(2) It was verified that the TCN network with added attention mechanism was better than the standard TCN network and LSTM network, which provided a new way to compensate for the error of MEMS gyro. Moreover, it was also verified that the compensation method of TCN network was more reasonable than that of LSTM network. By adding the attention mechanism, the model we proposed can focus on the temporal data being more decentralized rather than concentrating on the part of the sequence.
(3) By using the particle swarm optimization algorithm to estimate the Kalman filter parameters, the noise standard deviation reduction of Kalman filter parameter estimation was more satisfactory than that of the ordinary ARMA model. The calculation process was also more straightforward, and the curve fluctuations were relatively small. Compared to the original data, the noise standard deviation of the filtering effect of the combined model proposed in this paper decreased by 77.81% and 76.44% on the *x* and *y* axes, respectively. Additionally, the combined model reduced the noise effect by nearly three times compared to the traditional ARMA-KF filtering model, making the effect of the sensor more stable and effective.

In subsequent experiments, we shall perform dynamic experiments to obtain the MEMS gyroscope output, write the trained neural network model into the xPC module of the host computer for online real-time filtering, and build a platform to validate its practical engineering applications.

## Figures and Tables

**Table 1:** Details of Conv-DAE model.

Layer	Kernel Number	Kernel Size	Stride	Activation Function	Output Size	Padding
Conv1D	8	5	1	Relu	20 × 8	Same
MaxPooling	—	2	—	—	10 × 8	—
Conv1D	16	5	1	Relu	10 × 16	Same
MaxPooling	—	2	—	—	5 × 16	—
Conv1D_Trans	16	5	1	Relu	5 × 16	Same
UpSampling1D	—	2	—	—	10 × 16	—
Conv1D_Trans	8	5	1	Relu	10 × 8	Same
UpSampling1D	—	2	—	—	20 × 8	—
Conv1D_Trans	1	5	1	—	20 × 1	Same

**Table 2:** Equipment specifications of STIM300 gyroscope.

	Parameter	Normal
Gyro	Input Range	±400°/h
	Bandwidth (−3 dB)	262 Hz
	Bias Instability (Allan Variance @25 °C)	0.3°/h
	Angular Random Walk (Allan Variance @25 °C)	0.15°/√h
General	Sample Rate	≤2000 sample/s
	Power Supply	5.0±0.5 V
	Operating Temperature	−40 ℃≤T≤+85 ℃
	RS422 Transmission Bit Rate	921,600 bps

**Table 3:** Details of Normal-DAE.

Layer	Units	Activation Function	Output Size
Dense	128	Relu	20 × 128
Dense	64	Relu	20 × 64
Dense	32	Relu	20 × 32
Dense	64	Relu	20 × 64
Dense	128	Relu	20 × 128
Dense	1	Tanh	20 × 1

**Table 4:** Comparison of denoising autoencoder results.

Axis	Method	Noise Variance (×10^−4^(°/s)^2^)	Noise Standard Deviation (×10^−2^°/s)	Percentage
*X*-Axis	Raw	76.029	8.7194	—
	Normal-DAE	44.608	6.6789	76.59%
	**Conv-DAE**	**23.309**	**4.8280**	**55.37%**
*Y*-Axis	Raw	61.028	7.8120	—
	Normal-DAE	31.007	5.5684	71.28%
	**Conv-DAE**	**23.124**	**4.8088**	**61.56%**

**Table 5:** Basic settings for network training.

The output dimension of dense layer	1
Activation function of dense layer	Tanh
Batch size	512
Training epoch	100
TCN Kernel Size	4
TCN No. Blocks	1
TCN Padding	Causal

**Table 6:** MultiTCN architectures depending on the input data step (number of hidden layers = 4).

Input Data Step	MultiTCN Model Architecture		Noise Standard Deviation (×10^−2^°/s)	Time/Epoch
	Kernel Number	Dilations		
5	64	[1,2,4,8,16]	—	—
10	64	[1,2,4,8,16]	4.7156	41 s
15	64	[1,2,4,8,16]	4.6185	65 s
**20**	**64**	**[1,2,4,8,16]**	**4.5121**	**80 s**
25	64	[1,2,4,8,16]	4.5910	101 s
30	64	[1,2,4,8,16]	4.7347	140 s
40	64	[1,2,4,8,16]	4.8120	176 s

**Table 7:** MultiTCN architectures depending on the Kernel number (number of hidden layers = 4).

MultiTCN Model Architecture		Input Data Step	Noise Standard Deviation (×10^−2^°/s)	Time/Epoch
Kernel Number	Dilations			
16	[1,2,4,8,16]	20	—	—
32	[1,2,4,8,16]	20	4.8256	23 s
64	[1,2,4,8,16]	20	4.5121	80 s
**128**	**[1,2,4,8,16]**	**20**	**4.4920**	**116 s**
256	[1,2,4,8,16]	20	—	—

**Table 8:** MultiTCN architectures depending on the dilations (number of hidden layers = 4).

MultiTCN Model Architecture		Input Data Step	Noise Standard Deviation (×10^−2^°/s)	Time/Epoch
Dilations	Kernel Number			
**[1,2,4]**	**128**	**20**	**4.4737**	**61 s**
[1,2,4,8]	128	20	4.8256	83 s
[1,2,4,8,16]	128	20	4.4920	116 s

**Table 9:** MultiTCN architectures depending on number of hidden layers.

Number of Hidden Layers	MultiTCN Model Architecture		Noise Standard Deviation (×10^−2^°/s)	Time/Epoch
	Kernel Number	Dilations		
1	128	[1,2,4]	—	—
2	128	[1,2,4]	—	—
3	128	[1,2,4]	4.6564	55 s
**4**	**128**	**[1,2,4]**	**4.4737**	**61 s**
5	128	[1,2,4]	4.6110	85 s
6	128	[1,2,4]	4.7920	139 s
7	128	[1,2,4]	—	—

**Table 10:** Details of MultiTCN-Attention model.

Layer	MultiTCN-Attention Model Architecture				
	Kernel Number	Kernel Size	No. Blocks	Dilations	Padding
TCN1	128	4	1	[1,2,4]	causal
TCN2	128	4	1	[1,2,4]	causal
TCN3	128	4	1	[1,2,4]	causal
TCN4	128	4	1	[1,2,4]	causal
Attention	unit = 20				
Dense	unit = 1, activation = tanh				

**Table 11:** Comparison of *X*-axis results between MultiTCN-Attention and other neural networks.

Axis	Method	Noise Variance (×10^−4^(°/s)^2^)	Noise Standard Deviation (×10^−2^°/s)	Percentage
*X*-Axis	Raw	76.029	8.7194	—
	Conv-DAE	23.309	4.8280	55.37%
	LSTM	21.786	4.6675	53.53%
	MultiTCN	20.014	4.4737	51.31%
	**MultiTCN-Attention**	**13.317**	**3.6493**	**41.85%**

**Table 12:** Comparison of *Y*-axis results between MultiTCN-Attention and other neural networks.

Axis	Method	Noise Variance (×10^−4^(°/s)^2^)	Noise Variance (×10^−2^°/s)	Percentage
*Y*-Axis	Raw	61.028	7.8120	—
	Conv-DAE	23.124	4.8088	61.56%
	LSTM	21.362	4.6219	59.16%
	MultiTCN	16.271	4.0337	51.63%
	**MultiTCN-Attention**	**10.821**	**3.2895**	**42.11%**

**Table 13:** Details of ARMA-KF model.

Method	Φ	B	H	Q	R
*X*-Axis Raw-ARMA-KF	$\left[\begin{array}{cccc} 0.0497 & 0.7058 & 0.0131 & 0\\ 1 & 0 & 0 & 0\\ 0 & 1 & 0 & 0\\ 0 & 0 & 1 & 0 \end{array}\right]$	$\left[\begin{array}{cccc} 1 & -0.0534 & -0.6982 & 0\\ 0 & 0 & 0 & 0\\ 0 & 0 & 0 & 0\\ 0 & 0 & 0 & 0 \end{array}\right]$	$\left[\begin{array}{cccc} 1 & 0 & 0 & 0 \end{array}\right]$	$\left[\begin{array}{cccc} 0.0076 & 0 & 0 & 0\\ 0 & 0.0076 & 0 & 0\\ 0 & 0 & 0.0076 & 0\\ 0 & 0 & 0 & 0.0076 \end{array}\right]$	0.0079
*Y*-Axis Raw-ARMA-KF	$\left[\begin{array}{cccc} -0.4143 & -0.7462 & 0 & 0\\ 1 & 0 & 0 & 0\\ 0 & 1 & 0 & 0\\ 0 & 0 & 1 & 0 \end{array}\right]$	$\left[\begin{array}{cccc} 1 & 0.4105 & 0.7526 & 0\\ 0 & 0 & 0 & 0\\ 0 & 0 & 0 & 0\\ 0 & 0 & 0 & 0 \end{array}\right]$	$\left[\begin{array}{cccc} 1 & 0 & 0 & 0 \end{array}\right]$	$\left[\begin{array}{cccc} 0.0061 & 0 & 0 & 0\\ 0 & 0.0061 & 0 & 0\\ 0 & 0 & 0.0061 & 0\\ 0 & 0 & 0 & 0.0061 \end{array}\right]$	0.0062

**Table 14:** Details of PSO-KF model.

Axis	Method	Φ	H	Q	R
*X*-Axis	Raw-PSO-KF	0.8687	0.9617	0.1283	0.5465
	MultiTCN-Attention-PSO-KF	0.8871	0.9502	0.0343	0.7973
*Y*-Axis	Raw-PSO-KF	0.8689	0.9618	0.1284	0.5466
	MultiTCN-Attention-PSO-KF	0.8239	0.9217	0.0279	0.7547

**Table 15:** Comparison of Kalman filtering results in the *X*-axis.

Axis	Method	Noise Variance (×10^−4^(°/s)^2^)	Noise Standard Deviation (×10^−2^°/s)	Percentage
*X*-Axis	Raw	76.029	8.7194	—
	Raw-ARMA-KF	33.312	5.7717	66.19%
	Raw-PSO-KF	12.376	3.5179	40.35%
	MultiTCN-Attention	13.317	3.6493	41.85%
	**MultiTCN-Attention-PSO-KF**	**3.743**	**1.9348**	**22.19%**

**Table 16:** Comparison of Kalman filtering results in the *Y*-axis.

Axis	Method	Noise Variance (×10^−4^(°/s)^2^)	Noise Standard Deviation (×10^−2^°/s)	Percentage
*Y*-Axis	Raw	61.028	7.8120	—
	Raw-ARMA-KF	30.089	5.4854	70.22%
	Raw-PSO-KF	9.935	3.1520	40.34%
	MultiTCN-Attention	10.821	3.2895	42.11%
	**MultiTCN-** **Attention-PSO-KF**	**3.3878**	**1.8406**	**23.56%**
